# Evaluation of the Effects of Lipoxin A4 and Resolvin D1 on the Severity of Transient Tachypnea of the Newborn: A Prospective Study

**DOI:** 10.3390/children12101421

**Published:** 2025-10-21

**Authors:** Emrah Çığrı, Funda Çatan İnan, Sedat Gülten, Mehmet Akif Bildirici, Ayşe Ece Gökkaya, Metin Asıleren, Fethiye Yıldız, Hilmi Onur Kabukcu

**Affiliations:** 1 Department of Paediatrics, Faculty of Medicine, Kastamonu University, 37150 Kastamonu, Turkey; 2 Department of Biostatistics and Medical Informatics, Faculty of Medicine, Bilecik Seyh Edebali University, 11230 Bilecik, Turkey; 3 Department of Biochemistry, Faculty of Medicine, Kastamonu University, 37150 Kastamonu, Turkey

**Keywords:** transient tachypnea of the newborn, lipoxin, resolvin

## Abstract

**Highlights:**

Our article examines the effects of SPMs on transient neonatal tachypnea, a common condition in newborns. This study is the first clinical study conducted in this area.

**What are the main findings?**

- LXA4 and RvD1 levels were found to be lower in infants with severe TTN.
- RvD1 and PLR are significant in predicting infants with severe TTN.

**What is the implication of the main finding?**

- Infants with high LXA4 and RvD1 levels have milder TTN.
- RvD1 and PLR are specific and sensitive biomarkers in predicting infants with severe TTN.

**Abstract:**

**Objective:** Transient tachypnea of the newborn (TTN) is a common condition observed in neonates. Since its management often requires intensive care and leads to maternal–infant separation, it is a major source of parental concern. The present study aimed to evaluate the effects of lipoxin A4 and resolvin D1 on the clinical course of TTN and to determine whether complete blood count parameters could serve as predictors of disease severity. **Materials and Methods:** A total of 62 neonates admitted to the neonatal intensive care unit with a diagnosis of TTN were included. According to Silverman scoring, infants were divided into a mild group (n = 31) and a severe group (n = 31). Lipoxin A4 and resolvin D1 levels, together with complete blood count parameters, were compared between the two groups. Logistic regression and receiver operating characteristic (ROC) curve analyses were performed to assess the predictive value of these parameters for the clinical course. **Results:** Serum lipoxin A4 (*p* = 0.005) and resolvin D1 (*p* = 0.002) levels were significantly higher in the mild group compared with the severe group, whereas the neutrophil-to-lymphocyte ratio (*p* = 0.044) and platelet-to-lymphocyte ratio (*p* = 0.027) were significantly lower. Resolvin D1 and the platelet-to-lymphocyte ratio were identified as significant predictors of severe disease. In predicting a mild course, lipoxin A4 demonstrated the highest sensitivity (80.6%), while resolvin D1 exhibited the highest specificity (87.1%). **Conclusions:** Lipoxin A4 and resolvin D1 appear to play a protective role in preventing severe clinical progression of transient tachypnea of the newborn.

## 1. Introduction

Transient tachypnea of the newborn (TTN) is a common cause of respiratory distress in term and late preterm infants, with an estimated incidence of 4–5% [1,2]. Low birth weight, maternal diabetes, maternal asthma, male sex, perinatal asphyxia, macrosomia, cesarean delivery, and prematurity are some of several perinatal risk factors linked to TTN [3].

With clinical symptoms, TTN is typically seen as a benign, self-limiting illness, typically resolving within 48–72 h. However, in some cases, it may be linked to serious problems including pneumothorax and persistent pulmonary hypertension [4]. There is no specific treatment for TTN; supportive care, including adequate oxygenation and nutritional support, is usually sufficient [5].

Immune cells use stereoselective conversion of essential fatty acids, including docosahexaenoic acid (DHA), arachidonic acid (AA), and eicosapentaenoic acid (EPA), to form bioactive lipids referred to as specialized pro-resolving mediators (SPMs). SPMs are categorized into four main families: lipoxins, resolvins, maresins, and protectins [6]. Resolvin D1 (RvD1) and Lipoxin A4 (LXA4) are synthesized through sequential lipoxygenase pathways from AA and DHA precursors, respectively [7].

This study is the first to evaluate the relationship between TTN and SPMs. The purpose of this investigation was to assess the potential role of SPMs in modulating disease severity in neonates with TTN. Additionally, we investigated changes in complete blood count (CBC) parameters according to the severity of TTN.

## 2. Materials and Methods

GPower version 3.1 was employed to determine the size of sample. The minimum sample size needed to attain statistical power was determined to be 52 newborns, according to a 2-tailed alpha error of 0.05, a power of 0.80, and an effect size of 0.8. However, considering potential data loss, 62 infants in total were ultimately observed and analyzed in the study.

This research included neonates born at ≥35 weeks of gestation via normal spontaneous vaginal delivery (NSVD) or cesarean section (C/S) between December 2024 and August 2025 in our hospital, who were subsequently accepted to the neonatal intensive care unit (NICU) with TTN diagnoses. The following criteria were used to establish the TTN diagnosis [8]:

1. Presence of respiratory distress symptoms such as grunting, tachypnea, nasal flaring, and chest retractions that start during the first six hours of life and last for a minimum of twelve hours.
2. A chest radiograph showing at least one of the following features: symmetrical perihilar congestion, fluid in the interlobar fissures (fissure sign), prominent pulmonary vasculature, and hyperinflation of the lungs.
3. Elimination of additional possible reasons for respiratory distress, including metabolic disorders, congenital pneumonia, surfactant deficiency, congenital heart disease, and meconium aspiration syndrome.

Neonates diagnosed with TTN were assessed using the Silverman Scoring System. Infants with a score of <7 were classified as the mild group, while those with a score of ≥7 were classified as the severe group. Silverman score evaluation was performed by a single blinded neonatologist. The Silverman Scoring System is defined as follows [9]:

1. Upper chest movement:
  ○ Synchronous: 0 points
  ○ Delayed inspiration: 1 point
  ○ See-saw movement: 2 points
2. Lower chest retraction:
  ○ None: 0 points
  ○ Barely visible: 1 point
  ○ Easily visible: 2 points
3. Xiphoid retraction:
  ○ None: 0 points
  ○ Barely visible: 1 point
  ○ Easily visible: 2 points
4. Nasal flaring:
  ○ None: 0 points
  ○ Barely visible: 1 point
  ○ Easily visible: 2 points
5. Expiratory grunting:
  ○ None: 0 points
  ○ Audible with stethoscope: 1 point
  ○ Audible without stethoscope: 2 points

After obtaining informed consent from the relatives of neonates diagnosed with TTN who agreed to participate, a blood sample of 3 mL was collected from each infant at the 12th postnatal hour. Before being analyzed, the specimens went through a centrifuge for 15 min at 4000 rpm, and the supernatants were kept at −20 °C.

After reaching the target sample size, neonates in the mild and severe groups were compared on the basis of demographic characteristics (sex, birth weight, gestational age, and mode of delivery), levels of LXA4 and RvD1, neutrophil-to-lymphocyte ratio (NLR), platelet-to-lymphocyte ratio (PLR), and red cell distribution width (RDW). LXA4 and RvD1 levels were measured using a Biotek ELK800 reader (SunRedBio, Shanghai, China) and Biotek 50TS washer (SunRedBio, Shanghai, China) with the KC Junior program, while an automated hematological analyzer was used to measure NLR, PLR, and RDW parameters (XN-1000 Hematology Analyzer, Sysmex Corporation, Tokyo, Japan).

### 2.1. Exclusion Criteria

1. Infants with respiratory distress symptoms lasting less than 12 h;
2. Infants born at 34 weeks gestation or less;
3. Infants with congenital heart disease during prenatal follow-up;
4. Infants without an informed consent form.

### 2.2. Statistical Analysis

Version 26 of SPSS was used for all analyses. Skewness and kurtosis values were examined in order to determine whether the data was normal. Demographic and clinical characteristics of the Group1 (Silverman score < 7) and Group2 (Silverman score ≥ 7) were analyzed by comparison via *t*-test of independent samples. Receiver operating characteristic (ROC) curve analysis and the area under the curve (AUC) were employed to analyze the predictive performance of LXA4, RVD1, RDW, NLR, and PLR for Silverman score classification. Each variable’s values of sensitivity, specificity, and the Youden index were computed, and the maximum Youden index was used to establish the ideal cutoff point. A two-tailed *p*-value less than 0.05 was considered statistically significant.

## 3. Findings

This investigation analyzed a total of 62 infants with TTN, comprising 31 with mild TTN and 31 with severe TTN. The infants were included in the study consecutively. Regarding demographics, there were no statistically notable differences among the two groups (*p* > 0.05). Levels of LXA4 and RvD1 were notably greater in the mild group than that of the severe group (*p* < 0.05), while NLR and PLR values were significantly higher in the severe group than in the mild group (*p* < 0.05). There was no statistically notable variation among the groups regarding RDW (*p* > 0.05). Table 1 compares the two groups’ demographic data, LXA4 and RvD1 levels, and CBC parameters.

To assess the impacts of LXA4, RvD1, RDW, NLR, and PLR in predicting the severity of clinical course in TTN, a binary logistic regression analysis was used (Table 2). The model was found to be statistically significant (χ^2^(2) = 17.31, *p* < 0.001). It correctly classified 71% of the cases and explained 33.0% of the variance between groups (Nagelkerke R^2^). Among the variables, RvD1 (*p* = 0.005) and PLR (*p* = 0.022) were identified as significant predictors, with lower RvD1 levels and higher PLR values significantly increasing the likelihood of a severe TTN course. However, the variables LXA4, RDW, and NLR did not contribute significantly to the model.

**Figure 1:**
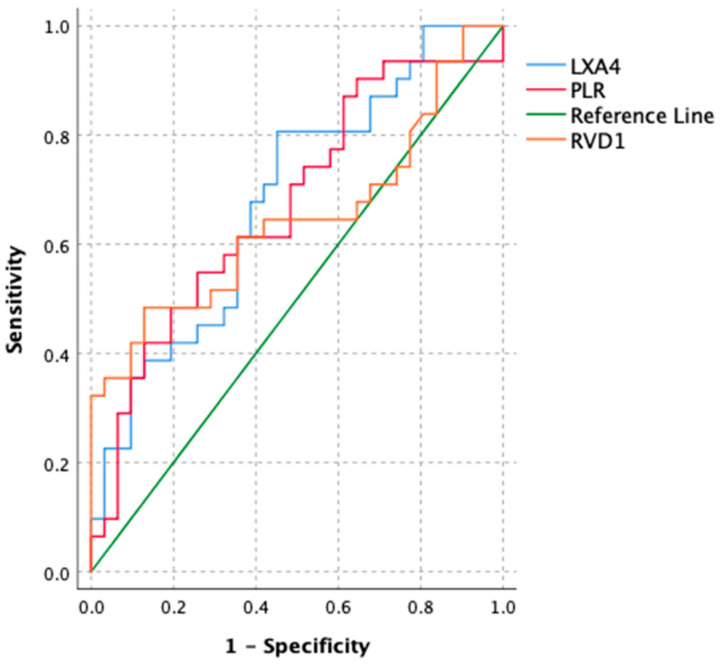
ROC curves of each variable for predicting group.

The results of the ROC analysis performed to ascertain the cutoff, specificity, and sensitivity values of LXA4, RvD1, and the PLR in predicting a milder course of TTN are presented in Figure 1 and Table 3. Among the tested biomarkers, LXA4 demonstrated the highest diagnostic accuracy, with an AUC value of 0.682 (*p* = 0.007). At a cutoff value of 0.678, its sensitivity was found to be 0.806, specificity 0.548, and the Youden index 0.354. RvD1 showed an AUC of 0.646 (*p* = 0.043), with a lower sensitivity of 0.484 but a higher specificity of 0.871 at the cutoff value of 1.22, yielding a Youden index of 0.355. The PLR demonstrated a moderate discriminative performance with an AUC of 0.671 (*p* = 0.013); at the cutoff value of 61.89, it showed a sensitivity of 0.548, specificity of 0.742, and a Youden index of 0.290. These findings suggest that LXA4 provides the best overall balance between sensitivity and specificity in identifying infants likely to experience a milder course of TTN, whereas RvD1 offers higher specificity in identifying these cases.

## 4. Discussion

In this investigation, we aimed to analyze the relationship between disease severity and the levels of LXA4 and RvD1 in newborns diagnosed with TTN and monitored in the NICU. We found that LXA4 and RvD1 have a protective effect against TTN, and that higher levels of these specialized SPMs were associated with milder disease courses. Furthermore, we observed that lower RvD1 levels and higher PLR values significantly increased the likelihood of severe TTN. Among the biomarkers studied, LXA4 demonstrated the highest sensitivity in predicting a mild disease course, while RvD1 showed the highest specificity.

According to earlier research, preterm babies born in an intrauterine environment that promotes inflammation are much more likely to experience complications like retinopathy of prematurity, necrotizing enterocolitis, intraventricular hemorrhage, periventricular leukomalacia, and bronchopulmonary dysplasia, compared with those developing in a normal intrauterine environment [10,11]. SPMs have been shown to exert anti-inflammatory impacts by preventing the synthesis of chemokines and pro-inflammatory cytokines, regulating neutrophil chemotaxis, and promoting the phagocytosis of apoptotic cells and debris by macrophages through G protein-coupled receptors [12,13]. Similarly, Tsai et al. [14] reported significantly lower plasma LXA4 levels in septic patients compared with healthy controls. These findings support the role of SPMs as critical mediators in neonatal health, particularly through their ability to regulate intrauterine inflammation.

Although the exact pathophysiology of TTN is not completely comprehended, delayed alveolar fluid clearance (AFC) is thought to be the underlying mechanism [15]. Alveolar epithelial type. I and II cells coordinate AFC through amiloride-sensitive sodium channels (ENaC) and Na, K-ATPase pumps to maintain alveolar homeostasis [16,17,18]. RvD1 has been reported to enhance ENaC and Na, K-ATPase activity, thus promoting AFC [19]. Additionally, Su et al. [20] and Perkins et al. [21] demonstrated that resolvin receptor agonists increased AFC under physiological conditions via a cAMP-dependent mechanism. Yang et al. [22] argued that administration of LXA4 in mice significantly inhibited the synthesis of pro-inflammatory cytokines such tumor necrosis factor-α and interleukin-6, resulting in reduced pulmonary edema and enhanced AFC. Recent studies [22,23,24] have further confirmed the potential of LXA4 and RvD1 in preserving lung function by regulating AFC in acute respiratory distress syndrome. In line with this literature, we also observed that greater values of LXA4 and RvD1 were linked with milder TTN, which may be explained by more efficient regulation of AFC mediated by these lipid mediators.

SPMs have a pivotal role in both the beginning and end of inflammation in pulmonary diseases [25]. Studies have shown that patients with severe asthma have significantly lower levels of SPMs in comparison with healthy controls [26,27]. In a neonatal mouse model of hyperoxia-induced lung injury, mice with higher LXA4 and RvD1 levels exhibited significantly reduced pulmonary inflammation and improved alveolarization compared with those with lower levels [28,29]. Similarly, Higgins et al. [30] demonstrated that LXA4 played a protective role in cystic fibrosis by reducing the invasion of *Pseudomonas aeruginosa* into bronchial epithelium. Other studies have shown that LXA4 significantly reduces allergic pleural eosinophil infiltration and inhibits edema and neutrophilia associated with allergic reactions [31]. Additionally, recent research points to a role for SPMs in the pathogenesis of COVID-19. A study demonstrated significantly reduced expression ofiiALOX5, the gene encoding 5-lipoxygenase (5-LO)—an enzyme critical for LXA4 synthesis—in airway macrophages and dendritic cells of patients with severe COVID-19 in comparison with healthy people, indicating that impaired biosynthesis of 5-LO-derived SPMs may be involved in SARS-CoV-2 pathogenesis [32]. Therefore, LXA4 and RvD1 may provide protection not only against TTN and bronchopulmonary dysplasia in the neonatal period, but also against asthma, pneumonia, and chronic lung disease later in life.

In humans, omega-3 supplementation has been found to increase circulating SPM levels [33]. Maternal supplementation with 600–800 mg/day of omega-3 during pregnancy was linked to increased SPM levels and reductions in preterm birth [34], intrauterine growth restriction [35], and NICU admission rates [36]. It has also been linked to a reduction in allergic conditions such as atopic dermatitis [37,38], food allergies [39], and asthma [40] in infants. Furthermore, supplementation has been shown to enhance antibody responses to routine childhood vaccinations [39] and reduce both the incidence and length of respiratory infections in infants under 6 months [41]. Weiss et al. [42] reported that breast milk contains significantly higher concentrations of LXA4 and D- and E-series resolvins during the first month postpartum compared to adult plasma levels. The protective role of SPMs in retinal injury is also well-established, with evidence supporting their potential as therapeutic agents for retinopathy of prematurity [43]. Connor et al. [44] demonstrated that administration of low-dose protectin and RvD1 in mice provided significant protection against neovascularization and vaso-obliteration. These findings imply that taking omega-3 supplements while pregnant and the promotion of breastfeeding could increase neonatal SPM levels, offering protection against a range of neonatal and childhood health issues.

In inflammatory and infectious diseases, lymphocyte counts tend to decrease while neutrophil counts increase, leading to elevated NLR values. Infections also stimulate cytokine production, which enhances platelet production and release into circulation, thereby increasing PLR [45]. Zheng et al. [46] reported significantly higher NLR values in children with severe pneumonia compared with healthy controls. Huang et al. [47] found NLR to be a reliable prognostic marker in sepsis, with higher NLR values indicating poorer outcomes. Bolat et al. [48] reported that both RDW and NLR at 72 h postnatally were effective in distinguishing TTN cases from healthy neonates and in predicting disease severity. In line with previous research, our investigation also discovered that infants with severe TTN had significantly greater NLR and PLR scores than those with milder forms, with PLR proving to be a useful predictor of disease severity. These observations support the view that TTN involves an inflammatory process, with biomarkers such as NLR and PLR increasing in parallel with disease severity.

## 5. Conclusions

High levels of LXA4 and RvD1 appear to contribute to a milder clinical course of TTN. Given their protective role against numerous neonatal and childhood disorders, the incidence and severity of TTN and other neonatal complications may be reduced through targeted pharmacological interventions, such as increasing SPM levels through maternal omega-3 supplementation, and effective perinatal strategies, such as promoting breastfeeding. Interventional studies with larger sample sizes are needed to obtain more definitive data.

## 6. Limitations

The most important limitations of our study are that it was conducted in a single center, the sample size was relatively small, and there was no healthy control group in our study. The prenatal omega-3 supplements taken by the mothers participating in the study may have affected the SPM levels of their babies. This is another limitation of our study.

## Figures and Tables

**Table 1:** Comparison of the demographic and clinical characteristics of the groups.

	Mild Group (n = 31)	Severe Group (n = 31)	*p*
Gestational Week	37.16 ± 1.46	36.84 ± 1.37	0.374
Delivery mode, n (%) C/S NSVD	15 (48.1) 16 (51.9)	24 (77.4) 7 (22.6)	0.025
Sex, n (%) Male Female	19 (61.3) 12(38.7)	16 (51.6) 15 (48.4)	0.365
Birth Weight (gram)	3152.58 ± 229.47	3094.19 ± 201.92	0.292
LXA4 (ng/mL)	1.11 ± 0.60	0.73 ± 0.38	0.005
RVD1 (ng/mL)	1.40 ± 0.81	0.86 ± 0.38	0.002
RDW (%)	17.05 ± 1.31	17.62 ± 1.69	0.138
NLR	1.29 ± 0.86	1.91 ± 1.44	0.044
PLR	50.76 ±21.77	65.72 ± 29.7	0.027

C/S: Cesarean section, NSVD: Normal spontaneous vaginal delivery, LXA4: Lipoxin A4, RvD1: Resolvin D1, RDW: Red cell distribution width, NLR: Neuthrophil/lymphocyte ratio, PLR: Platelet/lymphocyte ratio.

**Table 2:** Logistic regression analysis for prognostic factors among infants.

Variables	β	SE	*p*	OR	95% CI for OR	
					Lower	Upper
Constant	3.493	1.090	0.001	32.898		
RVD1	−1.731	0.621	0.005	0.177	0.052	0.598
PLR	−0.028	0.012	0.022	0.972	0.949	0.996

RvD1: Resolvin D1, PLR: Platelet/lymphocyte ratio.

**Table 3:** Cutoff values and areas under the ROC curves of each variable for predicting group.

Variables	Cutoff	Sensitivity	Specificity	Youden Index	AUC (95% CI)	*p*
LXA4	0.678	0.806	0.548	0.354	0.682 (0.549–0.814)	0.007
RVD1	1.22	0.484	0.871	0.355	0.646 (0.505–0.814)	0.043
PLR	61.89	0.548	0.742	0.290	0.671(0.536–0.807)	0.013

LXA4: Lipoxin A4, RvD1: Resolvin D1, PLR: Platelet/lymphocyte ratio.

## Data Availability

https://akgunhbyseah.kastamonusaglik.gov.tr/hbys-web/gen/anasayfa.htm, accessed on 1 September 2025.

## References

[1] Hibbard J.U., Wilkins I., Sun L., Gregory K., Haberman S., Hoffman M., Kominiarek M.A., Reddy U., Bailit J., Consortium on Safe Labor (2010). Respiratory morbidity in late preterm births. JAMA.

[2] Hansen A.K., Wisborg K., Uldbjerg N., Henriksen T.B. (2008). Risk of respiratory morbidity in term infants delivered by elective caesarean section: Cohort study. BMJ.

[3] Ozkiraz S., Gokmen Z., Boke S.B., Kilicdag H., Ozel D., Sert A. (2013). Lactate and lactate dehydrogenase in predicting the severity of transient tachypnea of the newborn. J. Matern. Fetal Neonatal Med..

[4] Lakshminrusimha S., Keszler M. (2015). Persistent pulmonary hypertension of the newborn. Neoreviews.

[5] Bak S.Y., Shin Y.H., Jeon J.H., Park K.H., Kang J.H., Cha D.H., Han M.Y., Jo H.S., Lee K.H., Lee C.A. (2012). Prognostic factors for treatment outcomes in transient tachypnea of the newborn. Pediatr. Int..

[6] Dalli J. (2017). Does promoting resolution instead of inhibiting inflammation represent the new paradigm in treating infections?. Mol. Aspects Med..

[7] Recchiuti A., Serhan C.N. (2012). Pro-resolving lipid mediators (SPMs) and their actions in regulating miRNA in novel resolution circuits in inflammation. Front. Immunol..

[8] Kasap B., Duman N., Özer E., Tatlı M., Kumral A., Ozkan H. (2008). Transient tachypnea of the newborn: Predictive factor for prolonged tachypnea. Pediatr. Int..

[9] Hedstrom A.B., Gove N.E., Mayock D.E., Batra M. (2018). Performance of the Silverman Andersen Respiratory Severity Score in predicting PCO_2_ and respiratory support in newborns: A prospective cohort study. J. Perinatol..

[10] Eriksson L., Haglund B., Odlind V., Altman M., Ewald U., Kieler H. (2015). Perinatal conditions related to growth restriction and inflammation are associated with an increased risk of bronchopulmonary dysplasia. Acta Paediatr..

[11] Strunk T., Inder T., Wang X., Burgner D., Mallard C., Levy O. (2014). Infection-induced inflammation and cerebral injury in preterm infants. Lancet Infect. Dis..

[12] Olsen S.F., Secher N.J. (2002). Low consumption of seafood in early pregnancy as a risk factor for preterm delivery: Prospective cohort study. BMJ.

[13] Olsen S.F., Østerdal M.L., Salvig J.D., Weber T., Tabor A., Secher N.J. (2007). Duration of pregnancy in relation to fish oil supplementation and habitual fish intake: A randomized clinical trial. Eur. J. Clin. Nutr..

[14] Tsai W.H., Shih C.H., Yu Y.B., Hsu H.C. (2013). Plasma levels in sepsis patients of annexin A1, lipoxin A4, macrophage inflammatory protein-3α, and neutrophil gelatinase-associated lipocalin. J. Chin. Med. Assoc..

[15] Jain L., Eaton D.C. (2006). Physiology of fetal lung fluid clearance and the effect of labor. Semin. Perinatol..

[16] Flodby P., Kim Y.H., Beard L.L., Gao D., Ji Y., Kage H., Liebler J.M., Minoo P., Kim K., Borok Z. (2016). Knockout mice reveal a major role for alveolar epithelial type I cells in alveolar fluid clearance. Am. J. Respir. Cell Mol. Biol..

[17] Johnson M.D., Widdicombe J.H., Allen L., Barbry P., Dobbs L.G. (2002). Alveolar epithelial type I cells contain transport proteins and transport sodium. Proc. Natl. Acad. Sci. USA.

[18] Matalon S., O’Brodovich H. (1999). Sodium channels in alveolar epithelial cells: Molecular characterization, biophysical properties, and physiological significance. Annu. Rev. Physiol..

[19] Serhan C.N., Chiang N., Dalli J. (2017). New pro-resolving n-3 mediators bridge resolution of infectious inflammation to tissue regeneration. Mol. Aspects Med..

[20] Su X., Robriquet L., Folkesson H.G., Matthay M.A. (2006). Protective effect of endogenous beta-adrenergic tone on lung fluid balance in acute bacterial pneumonia in mice. Am. J. Physiol. Lung Cell. Mol. Physiol..

[21] Perkins G.D., McAuley D.F., Thickett D.R., Gao F. (2006). The beta-agonist lung injury trial (BALTI): A randomized placebo-controlled clinical trial. Am. J. Respir. Crit. Care Med..

[22] Yang Y., Cheng Y., Lian Q.Q., Yang L., Qi W., Wu D., Zheng X., Liu Y., Li W., Jin S. (2013). Contribution of CFTR to alveolar fluid clearance by lipoxin A4 via PI3K/Akt pathway in LPS-induced acute lung injury. Mediat. Inflamm..

[23] Wang Q., Lian Q.Q., Li R., Ying B.-Y., He Q., Chen F., Zheng X., Yang Y., Wu D.-R., Zheng S.-X. (2013). Lipoxin A4 activates alveolar epithelial sodium channel, Na/K-ATPase, and increases alveolar fluid clearance. Am. J. Respir. Cell. Mol. Biol..

[24] Wang Q., Zheng X., Cheng Y., Zhang Y.-L., Wen H.-X., Tao Z., Li H., Hao Y., Gao Y., Yang L.-M. (2014). Resolvin D1 stimulates alveolar fluid clearance through alveolar epithelial sodium channel and Na/K-ATPase via ALX/cAMP/PI3K pathway in LPS-induced acute lung injury. J. Immunol..

[25] Krishnamoorthy N., Abdulnour R.E., Walker K.H., Engstrom B.D., Levy B.D. (2018). Specialized pro-resolving mediators in innate and adaptive immune responses in airway diseases. Physiol. Rev..

[26] Levy B.D., Kohli P., Gotlinger K., Haworth O., Hong S., Kazani S., Israel E., Haley K.J., Serhan C.N. (2007). Protectin D1 is generated in asthma and dampens airway inflammation and hyperresponsiveness. J. Immunol..

[27] Planagumà A., Kazani S., Marigowda G., Haworth O., Mariani T.J., Israel E., Bleecker E.R., Curran-Everett D., Erzurum S.C., Calhoun W.J. (2008). Airway lipoxin A4 generation and lipoxin A4 receptor expression are decreased in severe asthma. Am. J. Respir. Crit. Care. Med..

[28] Rogers L.K., Valentine C.J., Pennell M., Velten M., Britt R.D., Dingess K., Zhao X., Welty S.E., Tipple T.E. (2011). Maternal docosahexaenoic acid supplementation decreases lung inflammation in hyperoxia-exposed newborn mice. J. Nutr..

[29] Ma L., Li N., Liu X., Shaw L., Calzi S.L., Grant M.B., Neu J.N. (2012). Arginyl-glutamine dipeptide or docosahexaenoic acid attenuate hyperoxia-induced lung injury in neonatal mice. Nutrition.

[30] Higgins G., Fustero Torre C., Tyrrell J., McNally P., Harvey B.J., Urbach V. (2016). Lipoxin A4 prevents tight junction disruption and delays colonization of cystic fibrosis bronchial epithelial cells by *Pseudomonas aeruginosa*. Am. J. Physiol. Lung Cell. Mol. Physiol..

[31] Fukunaga K., Kohli P., Bonnans C., Fredenburgh L.E., Levy B.D. (2005). Cyclooxygenase 2 plays a pivotal role in the resolution of acute lung injury. J. Immunol..

[32] Sahanic S., Löffler-Ragg J., Tymoszuk P., Hilbe R., Demetz E., Masanetz R.K., Theurl M., Holfeld J., Gollmann-Tepeköylü C., Tzankov A. (2021). The role of innate immunity and bioactive lipid mediators in COVID-19 and influenza. Front. Physiol..

[33] Souza P.R., Marques R.M., Gomez E.A., Colas R.A., De Matteis R., Zak A., Patel M., Collier D.J., Dalli J. (2020). Enriched marine oil supplements increase peripheral blood specialized pro-resolving mediators and reprogram host immune responses: A randomized placebo-controlled study. Circ. Res..

[34] Carlson S.E., Colombo J., Gajewski B.J., Gustafson K.M., Mundy D., Yeast J., Georgieff M.K., Markley L.A., Kerling E.H., Shaddy D.J. (2013). DHA supplementation and pregnancy outcomes. Am. J. Clin. Nutr..

[35] Emmett P.M., Jones L.R., Northstone K. (2015). Dietary patterns in the Avon Longitudinal Study of Parents and Children. Nutr. Rev..

[36] Nordgren T.M., Lyden E., Anderson-Berry A., Hanson C. (2017). Omega-3 fatty acid intake of pregnant women and women of childbearing age in the United States: Potential for deficiency?. Nutrients.

[37] Palmer D.J., Sullivan T., Gold M.S., Prescott S.L., Heddle R., Gibson R.A., Makrides M. (2012). Effect of n-3 long-chain polyunsaturated fatty acid supplementation in pregnancy on infants’ allergies in the first year of life: Randomized controlled trial. BMJ.

[38] Furuhjelm C., Warstedt K., Larsson J., Fredriksson M., Fagerås Böttcher M., Fälth-Magnusson K., Duchén K. (2009). Fish oil supplementation in pregnancy and lactation may decrease the risk of infant allergy. Acta Paediatr..

[39] Furuhjelm C., Warstedt K., Fageras M., Fälth-Magnusson K., Larsson J., Fredriksson M., Duchén K. (2011). Allergic disease in infants up to 2 years of age in relation to plasma omega-3 fatty acids and maternal fish oil supplementation. Pediatr. Allergy Immunol..

[40] Olsen S.F., Osterdal M.L., Salvig J.D., Mortensen L.M., Rytter D., Secher N.J., Henriksen T.B. (2008). Fish oil intake compared with olive oil intake in late pregnancy and asthma in the offspring: 16 y of registry-based follow-up from a randomized trial. Am. J. Clin. Nutr..

[41] Imhoff-Kunsch B., Stein A.D., Martorell R., Parra-Cabrera S., Romieu I., Ramakrishnan U. (2011). Prenatal docosahexaenoic acid supplementation and infant morbidity: Randomized controlled trial. Pediatrics.

[42] Weiss G.A., Troxler H., Klinke G., Rogler D., Braegger C., Hersberger M. (2013). High levels of anti-inflammatory and pro-resolving lipid mediators in human milk during the first month of lactation. Lipids Health Dis..

[43] Malamas A., Chranioti A., Tsakalidis C., Dimitrakos S.A., Mataftsi A. (2017). The omega-3 and retinopathy of prematurity relationship. Int. J. Ophthalmol..

[44] Connor K.M., SanGiovanni J.P., Lofqvist C., Aderman C.M., Chen J., Higuchi A., Hong S., Pravda E.A., Majchrzak S., Carper D. (2007). Increased dietary intake of omega-3 polyunsaturated fatty acids reduces pathological retinal angiogenesis. Nat. Med..

[45] Altas O.F., Kizilkaya M. (2021). The effects of neutrophil-lymphocyte ratio, platelet-lymphocyte ratio and prognostic markers in determining mortality in patients with pneumonia in intensive care. Medeni. Med. J..

[46] Zheng H.H., Xiang Y., Wang Y., Zhao Q.-S., Dai R. (2022). Clinical value of blood-related indexes in the diagnosis of bacterial infectious pneumonia in children. Transl. Pediatr..

[47] Huang Z., Fu Z., Huang W., Huang K. (2020). Prognostic value of neutrophil-to-lymphocyte ratio in sepsis: A meta-analysis. Am. J. Emerg. Med..

[48] Bolat F., Haspolat N.Y., Bolat G., Şahin M. (2021). Simple hematological markers in predicting the severity of transient tachypnea of newborn: New wine of old bottles. J. Trop. Pediatr..

